# Dimensionality and scale properties of the Edinburgh Depression Scale (EDS) in patients with type 2 diabetes mellitus: the DiaDDzoB study

**DOI:** 10.1186/1471-244X-11-141

**Published:** 2011-08-24

**Authors:** Evi SA de Cock, Wilco HM Emons, Giesje Nefs, Victor JM Pop, François Pouwer

**Affiliations:** 1 Department of Medical Psychology & Neuropsychology, Center of Research on Psychology in Somatic diseases (CoRPS), Tilburg University, Tilburg, The Netherlands; 2 Department of Developmental and Clinical Psychology, Tilburg University, Tilburg, The Netherlands; 3 Department of Methodology and Statistics, Tilburg University, Tilburg, The Netherlands

## Abstract

**Background:**

Depression is a common complication in type 2 diabetes (DM2), affecting 10-30% of patients. Since depression is underrecognized and undertreated, it is important that reliable and validated depression screening tools are available for use in patients with DM2. The Edinburgh Depression Scale (EDS) is a widely used method for screening depression. However, there is still debate about the dimensionality of the test. Furthermore, the EDS was originally developed to screen for depression in postpartum women. Empirical evidence that the EDS has comparable measurement properties in both males and females suffering from diabetes is lacking however.

**Methods:**

In a large sample (*N *= 1,656) of diabetes patients, we examined: (1) dimensionality; (2) gender-related item bias; and (3) the screening properties of the EDS using factor analysis and item response theory.

**Results:**

We found evidence that the ten EDS items constitute a scale that is essentially one dimensional and has adequate measurement properties. Three items showed differential item functioning (DIF), two of them showed substantial DIF. However, at the scale level, DIF had no practical impact. Anhedonia (the inability to be able to laugh or enjoy) and sleeping problems were the most informative indicators for being able to differentiate between the diagnostic groups of mild and severe depression.

**Conclusions:**

The EDS constitutes a sound scale for measuring an attribute of general depression. Persons can be reliably measured using the sum score. Screening rules for mild and severe depression are applicable to both males and females.

## Background

Patients with type 2 diabetes mellitus (DM2) have about a two-fold increased risk of major depression, affecting at least one in every ten diabetes patients [1-3]. Depression not only has a serious negative impact on the quality of life of diabetes patients [4], but is also associated with poorer glycemic control, worse cardiovascular outcomes, and an increased health care consumption [5-7]. Depression is particularly common in diabetes patients with co-morbidity [2,3,8] and is associated with higher levels of diabetes-specific emotional distress [9].

It has been shown that depression in diabetes patients can be successfully treated by means of cognitive behavioral therapy, anti-depressive medication, or a combination of both [10]. However, an important barrier to effective treatment is the generally low recognition rate of depression [11,12]. International clinical guidelines advocate screening for depression in patients with diabetes [13-15]. Results from studies in non-diabetes patients suggest that screening for depression *per se *does not improve outcome [16]. It is crucial that screening procedures are embedded in a managed care approach for co-morbid depression that includes the monitoring of depression outcomes [16,17].

A proxy for depression is the occurrence of depressive symptoms: subjects with high levels of depressive symptoms do not necessarily meet the criteria for a syndromal diagnosis, but are at high risk for developing full blown major depression [18]. Moreover, it has clearly been demonstrated that subjects with high levels of depressive symptoms also have a poor quality of life, an increased resource utilization pattern, and a worse outcome regarding all kinds of somatic parameters of chronic disease, including diabetes [4,19,20]. Because of the high incidence of major depression in subjects with high depressive symptoms, most screening programs for depression use self-rating instruments. These instruments are user- friendly and large numbers of patients at risk can be approached. Subsequently, patients with a high score are subject to a syndromal diagnostic interview. So far, only a few measures of depressive symptoms have been tested for use in diabetes patients [21-25].

Since it is important that reliable and validated screening tools of depressive symptoms are available for use in patients with DM2, the aim of this study is to investigate the measurement properties of the Edinburgh Depression Scale (EDS) [26,27]. The EDS is a widely used screening tool that is regarded as suitable for screening purposes in various patient groups. It only takes a few minutes to complete and does not include items on the somatic symptoms of depression, such that the scores will not be biased by somatic symptoms caused by the disease. Although the EDS has been successfully applied in several studies [*e.g.*, [28,29]], there are three important issues that need further elaboration.

Firstly, there is ambiguity in the literature as to whether the EDS measures one or multiple dimensions. Some studies found support for a one-dimensional model [30,31], whereas others for a multi-dimensional model, comprising dimensions relating to depression, anhedonia, and anxiety [32-35]. For a valid interpretation of the EDS scores, it is important that these have an unequivocal meaning and do not represent a mixture of distinct characteristics. In the latter case, it would be inappropriate to use sum scores and the use of EDS subscales should be recommended.

Secondly, the EDS was originally developed to measure depressive symptoms in postnatal women and was called the Edinburgh Postnatal Depression Scale [26]. In recent years, the EDS has become more widely used in other patient samples that include both males and females. However, in some instances, the response to an item may have a different meaning for males than for females. A classic example in the context of depression assessment is crying, which indicates a more severe level of depression in the case of males than of females [*e.g*., [36]]. Therefore, an important issue that should be empirically examined is whether the items apply similarly to males and females. If one or more items in the EDS are biased with respect to gender, the sum scores for males cannot be compared with those for females, and the items showing bias should be removed or different scoring rules for males and females should be applied.

Thirdly, in clinical practice the EDS is used as a screening instrument for respondents with elevated depressive symptoms [*e.g*., [28,29]]. For example, the EDS is routinely used to screen women with an increased risk of postpartum depression [37]. Commonly recommended cutoff scores [27,38,39] include those of 12 or 13 to indicate patients with major depression, while those from 9 to 11 indicate patients with mild depressive symptoms who are in need of further assessment. Once accurate cutoff scores (*i.e*., high sensitivity/specificity) have been derived, it can be useful from a clinical perspective to investigate how the diagnostic groups differ at an item level, and which items provide the most information regarding differences in depression levels in the vicinity of these cutoff points. This information can be used to determine which items are the main indicators for distinguishing between mildly and severely depressed respondents. Practitioners working with the EDS can focus on the symptoms described by these items and use them as important 'signals' to identify those respondents who are about to become mildly or severely depressed [*e.g*., [40]]. In this study, we examine the test and item properties of the EDS for commonly used cutoffs [27,38,39].

The present study addresses these three issues in a large sample of patients with type 2 diabetes mellitus. To accomplish our aims, we used confirmatory factor analysis (CFA; [41]) and item response theory (IRT; [42]). Since its initial development, CFA has been widely applied to assess dimensionality. During the last decades, IRT has become increasingly popular for studying the measurement properties of self-report scales and questionnaires in the context of psychological and clinical assessment [43]. In the present study, both parametric and non-parametric IRT models [44,45] will be used, which together provide a flexible framework for studying the dimensionality, item bias, and measurement properties of the EDS.

## Methods

### Participants

The methods and design of the DiaDDZoB (Diabetes, Depression, Type D personality Zuidoost-Brabant) Study have been described in detail elsewhere [46]. Briefly, 2,460 type 2 diabetes patients (82% of those considered for inclusion in the study) treated at 77 primary care practices in south-eastern Brabant, the Netherlands, were recruited for the baseline assessment during the second half of 2005 (M0). Of these patients, 2,448 (almost 100%) attended a baseline nurse-led interview, while 1,850 (75%) returned the self-report questionnaire that had to be completed at home. In addition, results from regular care laboratory tests and physical examinations were also used. The study protocol of the DiaDDZoB Study was approved by the medical research ethics committee of a local hospital: Máxima Medical Centre, Veldhoven (NL27239.015.09). In the present study, we only used data from participants who completed all the EDS items, resulting in a sample of 1,656 participants.

### Measures

*The Edinburgh Depression Scale (EDS)*. The EDS is a self-report questionnaire consisting of ten items (for item content see Table 1, columns 1 and 2) with four ordered response categories scored from 0 to 3. After recoding the reverse worded items, sum scores may range from 0 to 30; the higher the sum score, the higher the level of depression. In the present study, a Dutch version of the EDS was used. The EDS has been validated in various countries, including the Netherlands, using different methods [32,47-49]. When used as a screening instrument, the cutoff scores of 12/13 usually designate major depression, whereas scores from 9 to 11 indicate mild depression levels in need of further assessment [27,37].

**Table 1:** Descriptive item and scale statistics and results of confirmatory factor analyses

			Factor Loadings			
			
			CFA Polychoric ^1^	FI One-Factor Model^2^	Bifactor Model	
					
	Item Content	Item Mean (SD)	$\underset{-}{\beta }$		General Factor	Specific Factor
1	I have been able to laugh and see the funny side of things	0.37 (0.73)	.82	.69	.62	.63
2	I have looked forward with enjoyment to things	0.42 (0.82)	.81	.68	.61	.74
3*	I have blamed myself unnecessarily when things went wrong	1.06 (0.86)	.52	.51	.53	--
4	I have been anxious or worried for no good reason	0.90 (0.89)	.65	.64	.65	--
5*	I have felt scared or panicky for no very good reason	0.78 (0.83)	.70	.69	.71	--
6*	Things have been getting on top of me	0.81 (0.76)	.75	.74	.75	--
7*	I have been so unhappy that I have had difficulty sleeping	0.62 (0.80)	.80	.79	.80	--
8*	I have felt sad or miserable	0.53 (0.67)	.84	.83	.83	--
9*	I have been so unhappy that I have been crying	0.28 (0.53)	.74	.73	.73	--
10*	The thought of harming myself has occurred to me	0.09 (0.37)	.67	.67	.68	--

Sum score		5.86 (4.78)				

Reliability		.84^3^		.83	.83	

* item recoded in order that higher scores indicate higher levels of depression

^1^CFA Polychoric = Confirmatory Factor Analysis on Polychoric correlation matrix; ^2^FI One-Factor Model = Full-Information One-Factor Model; ^3^Cronbach's alpha

### Statistical Analyses

#### Item Response Theory

The core of IRT models is the set of item-response functions (IRF), which describe the relationship between item responses and the hypothesized latent attribute of interest. Within the IRT framework, a distinction can be made between parametric IRT approaches [50,51] and nonparametric IRT [52]. The difference between parametric and nonparametric IRT models is the way in which they define the shape of these cumulative IRFs. Parametric IRT models specify the IRF using a mathematical function. Nonparametric IRT models only assume a monotone increasing relationship between attribute and item responses, but do not require a parametric function. This property makes nonparametric IRT models excellent starting points in any IRT analysis, particularly for the purposes of (exploratory) dimensionality analysis and early identification of malfunctioning items.

For the nonparametric IRT analyses, we used Mokken's monotone homogeneity model (MHM) [52, Chap. 7] and for the parametric IRT analyses, Samejima's graded response model (GRM) [53], which are both suitable for analyzing ordered polytomous item responses (*i.e*., Likert items). Both the MHM and the GRM assume that only one single latent attribute underlies the responses (*i.e*., the assumption of unidimensionality) and that the association between item scores is solely explained by this single attribute (*i.e*., the assumption of local independence). To explain the differences between the IRFs under the MHM and GRM, some notation should be introduced. Therefore, let *M + *1 be the number of response options (*i.e*., *M *= 3 for the EDS) and *θ *denote the latent attribute of interest (*i.e*., *θ *represents depression in the EDS). Furthermore, let *X_j_*denote the item-score variable for item *j *and *X*_+ _the sum score. Under the MHM and GRM, each item is described by *M *cumulative IRFs, with the *m*th IRF describing the probability of scoring in category *m *or higher as a function of *θ*. The probability of answering *within *a particular category can easily be derived from the cumulative IRFs ([42], *p*. 99).

**Figure 1:**
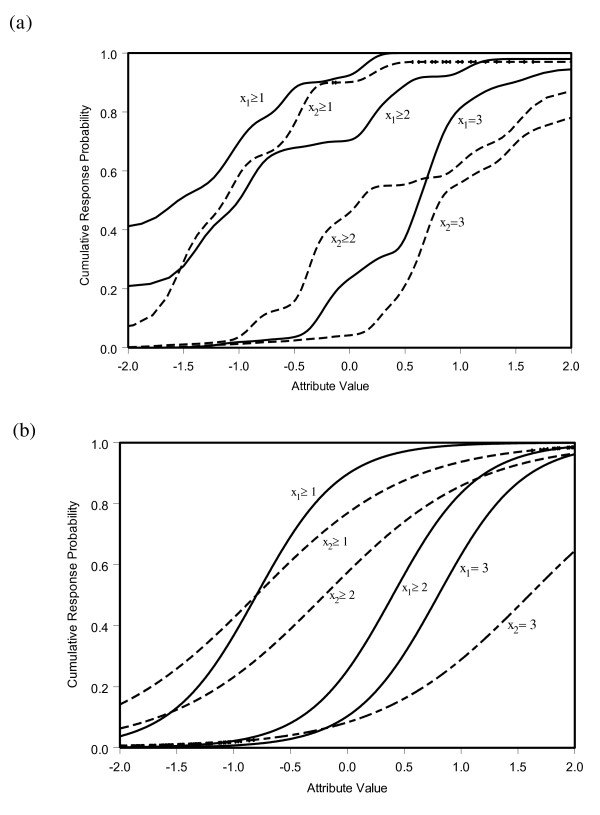
**Examples of cumulative item response functions (IRFs) under (*a*) Mokken's Homogeneity model and (*b*) the Graded Response Model**.

The MHM assumes that the IRFs are non-decreasing functions in *θ *(*i.e*., the monotonicity assumption), but within this restriction any shape is allowed. Examples of IRFs for two MHM items are provided in Figure 1A; the solid lines represent the IRFs of one item, and the dashed lines of another. Under Samejima's GRM, the IRFs are assumed to be logistic functions. Examples of IRFs under the GRM are provided in Figure 1B; the solid lines represent a highly discriminating item and the dashed lines a weakly discriminating one. The IRFs of an item *j *are defined by one common slope parameter (denoted by *a*) and *M *threshold parameters (denoted by *b_jm_*). The slope parameter *a*, indicates the discrimination power of an item; the higher the slope parameter *a*, the steeper the IRF and the better the item discriminates low *θ *values from high *θ *values. The thresholds *b_jm_*(*m *= 1,..., *M*) indicate how the item scores categorize the *θ *scale into *M *+ 1 groups and can be conceived as points on the latent *θ *scale where the item optimally discriminates high *θ *from low *θ *values.

The IRT approaches adopted in this study have several advantages compared to classical test theory ([54]) and Rasch analysis [55]. Firstly, Mokken models provide *empirical *justification for using sum scores as measurements of the underlying construct [52,56]. If a set of items fails to fit the MHM, respondents cannot be scaled on the underlying dimension by their sum scores. In classical test theory it is *assumed *that the sum scores are proper measurements of the underlying attribute, without testing this assumption empirically.

Secondly, the MHM and GRM are less restrictive than Rasch models and thus may be better able to describe the structure in the data and prevent researchers from dismissing items with adequate measurement properties for the wrong reasons. For example, the MHM - which was the most general measurement model used in the present study - only requires the IRFs to increase monotonically (Figure 1A). Items with monotone increasing functions are valid indicators of the underlying construct [56]. This means that, for valid measurement, IRFs do not necessarily have to conform to a logistic function, as required under the Rasch model. In addition, as in the case of the Rasch model, the GRM requires logistic functions, but unlike the Rasch model, the GRM permits varying slopes across the items (Figure 1B). Under the Rasch model, the IRFs would be parallel lines. The equal-slopes assumption in the Rasch model states that all the items in the questionnaire have the same discrimination power. In real data, this is often an unrealistic assumption and, as a result, a Rasch analysis may result in badly fitting items, not because the item is malfunctioning but because the item discrimination is *different *from the other items in the questionnaire.

### Issue 1: Is the EDS unidimensional?

*Exploratory dimensionality analysis*. To explore the dimensionality using IRT, we adopted Mokken scale analysis (MSA) [52], which is a scaling methodology based on the MHM. MSA has several advantages over exploratory factor analysis (EFA) on Pearson correlation matrices; see [57,58]. Firstly, MSA is based on less restrictive distributional assumptions than EFA and is therefore suitable for analyzing data from items with skewed score distributions (*e.g*., items that measure symptoms with a low prevalence in the population under study). With EFA, such items may lead to over-extraction of artificial difficulty factors that have no substantive meaning. Secondly, MSA explicitly takes into account the psychometric properties of items, such as the scalability, for uncovering unidimensional scales, whereas factor analysis only uses the inter-item correlations without testing whether items are psychometrically sound.

In an MSA, the dimensionality is explored using scalability coefficients, which are defined at the item level (denoted by *H_i_*) and the scale level (denoted by *H*). The item scalability coefficients *H_i _*indicate how well an item is related to other items in the scale and can be conceived as the nonparametric counterpart of an item loading in a factor analysis. The scale *H *value summarizes the item scale values into a single number and expresses the degree to which the sum score accurately orders persons on the latent attribute scale *θ *[52]. The higher the *H *value, the more accurately persons can be ordered using the sum score. To explore whether the items form one unidimensional scale, or several dimensionally distinct subscales, we used an automated item selection procedure (AISP) [52, Chap. 5, *pp*. 65 - 90]. This AISP sequentially clusters items into disjointed subsets of items, each representing one- dimensional attribute scales. The items are clustered under the restriction that the resulting scales and their constituent items yield scalability coefficients greater than a user-specified lower-bound value *c*. Therefore, this lower-bound *c *controls the minimum scalability level of the items to be included in the scale and must be chosen by the user. The following rules of thumb for choosing *c*-values are commonly used: .30 <*c *< .40 for finding weak scales, .40 <*c *< .50 for finding medium scales, and *c *> .50 for strong scales [see 52, p. 60]. The dimensionality can be revealed by evaluating the clusters produced by applying the AISP for different *c*-values increasing from .30 to .55 with steps of .05 [52, *p*. 81]. For unidimensional scales, the typical sequence of outcomes of the AISP with increasing *c*-values is that, first, all the items are in one scale, then one smaller scale is found, and finally, one or a few scales are found and several items are excluded [52, *p*. 81]. Within each step of the AISP, for each cluster it has to be evaluated whether its constituent items have non-decreasing IRFs in order to make sure that the scales fit the MHM. Items that have locally increasing IRFs violate the monotonicity assumption and should be removed from the cluster because they distort accurate person ordering using *X*_+_. All analyses were done with the Mokken Scale Analysis for Polytomous items (MSPWIN) program [59]. To facilitate dimensionality analysis, the results of MSA will be compared with those of a CFA on the polychoric correlation matrix in MPLUS5 [60].

### Issue 2: Are the items in the EDS unbiased with respect to gender?

An item is considered biased with respect to gender if the item parameters are significantly different for males and females. The phenomenon that parameters vary across groups is termed *differential item functioning *(DIF). If an item shows DIF, individuals from different groups, but with the same attribute levels, do not have the same response probabilities for that item. To test for DIF, we used IRT-based likelihood ratio tests (*e.g*., [61]) as implemented in the program IRTLRDIF2.0 [62]. To test for gender bias, the likelihood- ratio test compares the fit of two nested IRT models: a restricted model in which the item parameters are constrained to be identical between males and females (representing the null hypothesis of no gender bias), and a general model in which for one or more study items the item parameters may differ across the gender groups (alternative hypothesis of item bias). Significant differences in fit indicate gender bias for the study items and are inspected for clinical relevance.

To investigate the presence of DIF and to understand what kind of DIF it is, the IRTLRDIF program performs a series of statistical tests per study item. It starts with an overall test on the hypothesis that all parameters (*a *and *b*s) are equal (null hypothesis of no DIF) against the alternative that the parameters differ between males and females. A significant result means that slopes (*i.e*., discrimination power), thresholds (item popularity), or both, vary across the gender groups. Two additional tests are performed in the case of a significant overall test in order to facilitate further understanding of the type of DIF. Firstly, a test is carried out to see whether the slopes are equal without imposing restrictions on the thresholds. If the test on the slopes is not significant, the assumption of equal slopes is retained, which means that item bias only relates to gender-specific differences in the thresholds. This type of DIF is known as uniform bias. Secondly, the equality of the thresholds is tested, conditional on equal slopes. It may be noted that, when the slopes differ significantly, there is non-uniform DIF and a subsequent analysis of differences in thresholds has no meaningful interpretation [62, *p*. 10]

A critical assumption in IRT-based DIF analysis is that the respondents can be accurately matched on *θ*. This matching is based on a subset of the scale items (i.e., the anchor) and should not be contaminated by the presence of DIF items in it. Therefore, a DIF-free anchor must be identified [42, *p*. 259]. This is accomplished by means of an iterative purification process [63]. This approach starts with the complete set of items as the anchor and then DIF items are identified and removed from the anchor one-by-one. Each time an item is removed, the DIF analysis is repeated using the other non-DIF items as the anchor. This purification process proceeds until an item set remains that shows no DIF. To test for significance during each step of the purification process, we used a Bonferroni correction for the statistical tests in order to control the experiment-wise Type I error rate at the 5% level. More specifically, the Bonferroni correction sets the significance level (*α*) equal to 0.05/*K*, where *K *is the number of items that are subjected to a DIF analysis. Once a valid anchor of DIF-free items had been identified, a final DIF analysis was performed for each non-anchor item individually. Only the results of the final DIF analysis are reported.

### Issue 3: What are the measurement properties of the EDS for screening depression?

If the estimated IRT model fits the data adequately, the parameters from the IRT model can be used to explore and describe the measurement properties of the questionnaire and its constituent items. One of the valuable features of IRT modeling is the possibility of evaluating the test and item reliability at different ranges of the *θ*-scale [42]. This means that in IRT reliability is not conceived as a constant, but depends on the latent attribute value *θ*. In particular, IRT provides test and item information functions to examine the reliability at different ranges of *θ*; the higher the information function in a particular range of *θ*, the better the item can reliably discriminate low from high attribute levels within that *θ *range. Using information functions, Reise and Waller [43] found, for example, that for most clinical scales, individuals high on the attribute scale were measured more reliably than individuals low on the attribute scale.

To evaluate the screening properties of the EDS, we evaluated the information function around the latent cutoff points that differentiate between the diagnostic categories of non-depressed, mildly depressed, and severely depressed [27,38]. The latent cutoffs are those points on the *θ *scale that correspond with an expected score of *X*_+ _= 9 (cutoff score for screening mild depression) and *X*_+ _= 12 (cutoff score for screening severe depression). For each item, we computed the individual contribution to the total test information at each cutoff point. These individual contributions give an indication of which items are the most reliable indicators for distinguishing mild from no depression, and severe from mild depression (*e.g*., see [64]). We also evaluated the item-score profiles at the latent cutoff points. These profiles are the average item scores for respondents at the cutoffs, showing how the diagnostic groups differentiate at the individual item level.

## Results

### Descriptive Statistics

As shown in Table 2, the total study sample consisted of 1,656 patients (50% male; mean age 66 years). Overall, the participants were in relatively good glycemic control (mean HbA_1c_ 6.7%) and the majority was being treated with a combination of diet and oral agents. Males and females differed significant ly regarding several demographic and clinical variables (Table 2), but these differences have no implications for the present study. Item means and standard deviations are presented in Table 1 (column 3). The item means of all items were relatively low (range 0.09 to 1.06). Thus, in the present sample, item-score distributions were skewed, with the majority of participants scoring in the lower answer categories. In the sample of males, 9.8% had symptoms of mild depression (*i.e*., an EDS sum score in the range of 9 to 11) and 8.1% had symptoms of severe depression (*i.e*., scoring 12 or higher on the EDS). In the sample of females, the percentages of respondents with symptoms of mild and severe depression were 16.5% and 16.2%, respectively.

**Table 2:** Demographic, clinical, and psychological characteristics of male and female participants

	Male (*n *= 828)		Female (*n *= 828)	
**Demographic variables**				
Age (Mean, SD)	65	(10.0)	67	(10.6)**
Dutch or Caucasian ethnicity	98%	(799/815)	98%	(797/816)
Level of education				**
Low education	73%	(577/795)	87%	(688/793)
Average education	21%	(166/795)	9%	(72/793)
High education	6%	(51/795)	4%	(32/793)
Marital Status				**
Married	83%	(681/819)	68%	(558/819)
Single	8%	(69/819)	7%	(56/819)
Widow/widower	5%	(43/819)	22%	(181/819)
Other	3%	(26/819)	3%	(24/819)
**Medical history**				
Peripheral arterial disease	25%	(195/797)	22%	(172/800)
Bypass or angioplasty	17%	(140/807)	9%	(72/801)**
Myocardial infarction	15%	(123/804)	7%	(57/801)**
Stroke	8%	(62/806)	6%	(48/801)
Angina pectoris	13%	(100/798)	9%	(72/795)*
Kidney failure	3%	(27/799)	4%	(32/797)
Retinopathy	4%	(25/627)	5%	(28/594)
Foot problem	62%	(400/645)	64%	(417/653)
**Clinical variables**				
^HbA^1c ^(Mean, SD)^	6.7	(0.8)	6.7	(0.9)
BMI (Mean, SD)	28.1	(4.0)	29.9	(5.4)**
Cholesterol (Mean, SD)	4.3	(0.9)	4.7	(1.0)**
LDL (Mean, SD)	2.5	(0.8)	2.7	(0.8)**
HDL (Mean, SD)	1.2	(0.3)	1.3	(0.4)**
Systolic blood pressure (Mean, SD)	141.1	(17.8)	141.0	(18.4)
Diastolic blood pressure (Mean, SD)	78.4	(9.4)	77.8	(9.4)
Diabetes duration > 3 years	59%	(486/828)	57%	(475/828)
Diabetes treatment				
No treatment	1%	(8/823)	1%	(8/817)
Diet	18%	(148/823)	17%	(135/817)
Diet and oral agents	76%	(621/823)	76%	(617/817)
Diet and insulin	1%	(8/823)	2%	(12/817)
Diet, oral agents, and insulin	4%	(35/823)	6%	(45/817)
Other	0%	(3/823)	-	
**Psychological variables**				
Self-reported history of depression	8%	(60/800)	13%	(102/798)**

*Note*. Means of males and females are compared with independent samples *t*-tests, percentages are compared with χ^2^-tests. * *p *< .05, ** *p *< .001

### Results for Issue 1: Is the EDS unidimensional?

*Results for Exploratory Nonparametric IRT Analysis*. The results of the dimensionality analysis using MSA are presented in Table 3. For *c *= .30, all items were selected in one scale. Item *H_j_*values ranged from .36 to .56 and the *H *coefficient for the total scale was .46, which indicates medium scalability [52, *p*. 60]. With increasing values of *c*, more and more items left the first scale, a few other, smaller scales were formed, and more and more items became unscalable. According to Sijtsma and Molenaar [52, *p*. 81], such a pattern of item clustering is typical for unidimensional item sets. It can be seen that, for higher *c*-values (> .40), the AISP consistently found a two-item scale comprising items 1 and 2, which constituted a strong scale (Table 3, column 12). However, when the two items were included in the ten-item scale, they had *H*-values that were in same range as the *H*-values for all the other items. Such *H*-values under the one-factor solution suggest that the two items provide reliable information about the general depression dimension underlying all items, but also that the two items are strong measurements of a specific aspect of depression. This high association between these two items reveals local dependencies between them.

**Table 3:** Cluster solutions in the Automatic Item Selection Procedure for six levels of lower bound *c*

			Lower Bound *c*										
			
			.30	.40	.45			.50		.55		.60	
			
		Scale #	1	1	1	2	3	1	2	1	2	1	2
**Item**													

1.	laugh		.44	.47	.55	-	-	.55	-	.67	-	.72	-
2.	enjoyment		.44	.47	.54	-	-	.54	-	.64	-	.72	-
3.	blamed		.44	.44	-	.53	-	us	us	us	us	us	us
4.	anxious/worried		.36	Us	-	-	.47	-	.42	us	us	us	us
5.	scared/panicky		.45	.46	-	.53	-	us	us	us	us	us	us
6.	things get on top of me		.50	.52	.54	-	-	.54-	-	-	.57	-	.61
7.	difficulty sleeping		.51	.53	.55	-	-	.55	-	-	.59-	-	.61
8.	sad/miserable		.56	.58	.61	-	-	.61	-	.58	-	-	.63
9.	crying		.47	.48	.50	-	-	.50	-	-	.55	us	us
10.	thought of self harm		.44	.44	-	-	.47	-	.44	us	us	us	us

H			.46	.49	.55	.53	.47	.55	.53	.64	.57	.72	.62

*Note*. us = unscalable

To determine whether persons can be reliably ordered on the scale by means of *X*_+_, the monotonicity assumption was investigated by testing estimated IRFs for local decreases. Monotonicity was evaluated using item rest-score regressions, as implemented in the software package MSPWIN [59]. Several sample violations of monotonicity were found, but none of these was significant when tested at a 5% significance level. This means that the monotonicity assumption is supported by the data.

#### Confirmatory factor analysis (CFA)

To further study the dimensionality of the EDS, we used a CFA on the polychoric correlation matrix. Firstly, the one-factor model was fitted to the data. The standardized item- factor loadings for the one-factor CFA model are presented in Table 1 (column 4). Based on the factor loadings and the CFI and RMSEA (Table 4), the one-factor model with all ten items loading on the factor fitted well and can be accepted. However, inspection of the bivariate residuals showed positive residual association between items 1 and 2 (residual *r *= .169) and small or negative residuals between all other item pairs. This result indicates local dependence between items 1 and 2. To see whether the two locally-dependent items should be treated as a separate scale, we also fitted a correlated two-factor model, in which items 3 to 10 load on one factor and items 1 and 2 load on the other factor, and a one-factor model with items 3 to 10 (having removed items 1 and 2). Comparison of the fit indices for the two -factor model and the eight-item one-factor model with the ten-item one-factor model only showed minor improvements. However, the item-factor loadings of items 1 and 2 reduced from .82 and .81 to .64 each, when estimated separately in 9-item models (results not tabulated). This result indicates that the local dependence between the items led to inflated factor loadings. To summarize, CFA supports unidimensionality for the EDS, but identified local dependence between items 1 and 2.

**Table 4:** Model-fit indices polychoric correlations confirmatory factor analysis

Model	CFI^1^	TLI^1^	RSMSA^2^
Unidimensional (all 10 items)	.970	.981	.068
8-item scale (items 1&2 removed)	.985	.989	.058
Two-dimensional^3^	.974	.984	.063

*Notes*.

^1^CFI/TLI > .9 indicates reasonably good fit (Kline, 2005; *pp*. 137-141)

^2^RSMEA between 0.05 and 0.08 suggests reasonable fit (Kline, 2005; *pp*. 137-141)

^3^Two dimensional model; items 1 and 2 loaded on one factor, and items 3 to 10 on the other factor; factors were correlated.

#### Full Information Item Bifactor Analysis

Dimensionality analyses using MSA and CFA revealed local dependence and, as a result, did not yield convincing evidence that the EDS is truly unidimensional. Since unidimensionality is a critical assumption in IRT, additional analyses had to be carried out in order to verify to what extent observed deviations from unidimensionality may cause problems in subsequent IRT analysis of the EDS. To address this issue in greater detail, we performed a full-information item bifactor analysis (BFA), which can be conceived as a multidimensional IRT model [65,66]. In the bifactor model, all items load on a general factor, which in our case represents a broad construct of depression, and one or more item clusters each load on a specific factor representing a subdomain of depression. The specific factors are uncorrelated and do not correlate with the general factor. Comparison of the item factor loadings under the full information one-factor model and the factor loadings under the full information bifactor model provides diagnostic information about the usefulness of unidimensional IRT models in the presence of multidimensionality. If factor loadings for the one-factor model are close to those for the general factor under the bifactor model, unidimensional IRT modeling is justified [66].

Using BIFACTOR [67], we fitted the full-information one-factor model and bifactor model with items 1 and 2 loading on both the general and specific factor. The bifactor model fitted significantly better than the one-factor model (*χ*^2 ^(10) = 358.08; *p *< 0.001). For items 1 and 2, the factor loadings on the general factor in the bifactor model were about 1.1 times smaller than the corresponding loadings under the one-factor model (see Table 1, columns 5-6). No appreciable differences for the other items were found between the factor loadings under the one-factor model and bifactor model. Furthermore, the reliability of both the ten- item scale and the general factor under the bifactor model was 0.83 (Table 1, columns 5 and 6, last row).

To summarize, MSA, CFA, and BFA consistently showed that all items in the EDS load on the general attribute of interest. However, MSA and BFA identified local dependence between items 1 and 2, but the impact on the item loadings was small. When studying DIF, which focuses on the relative differences between males and females, such a small bias in parameter estimates can be safely ignored. Care should be taken in drawing conclusions when DIF is found only for items 1 and 2. However, the presence of local dependencies is more problematic for parameter estimation since it may spuriously inflate the estimated item discriminations [68]. To avoid biased estimates due to local dependency in the data, we used MULTILOG7 [69] and adopted a two-step procedure to obtain the parameter estimates not biased by local dependence (to be explained below).

### Results for Issue 2: Are the items in the EDS unbiased with respect to gender?

The purification process for finding a DIF-free anchor item set identified item 9 (*χ*^2 ^(4) = 92.1, *p *< .001), item 3 (*χ*^2 ^(4) = 27.2, *p *< .001), and item 4 (*χ*^2 ^(4) = 15.8, *p *= .003) (results not tabulated) as potentially biased items. The remaining seven items were used as anchor items in the final DIF analysis, and the other three items were individually tested for gender- related item bias. DIF analysis per item (see Table 5; columns 2 to 4) revealed gender-related DIF for item 3 (*blaming oneself*), item 4 (*anxious/worry*), and item 9 (*crying*). Additional χ^2 ^- tests for testing equality of the slope parameters between males and females were not significant for any of the items (Table 5; column 3). This means that the item slopes do not differ between males and females. We found significant DIF for items 3, 4, and 9 (Table 5; column 4) for the χ^2 ^-test for equality of thresholds. This means that the observed DIF for these items can be explained by differences in the thresholds between males and females.

**Table 5:** Results of testing for gender bias and estimated item parameters (standard error in italics) and item fit for females and males

Item	DIF			Estimated Item Parameters								Item Fit^1^
			
	Slopes and Thresholds equal	Slopes Equal	Thresholds equal	Females(*n *= 828)				Males(*n *= 828)				
			
	*χ*^2 ^(4)	*χ*^2 ^(1)	*χ*^2 ^(3)	*a*	*^b^*1	*^b^*2	*^b^*3	*a*	*^b^*1	*^b^*2	*^b^*3	*p-*value
1	7.6	0.8	6.8	1.46	0.81	1.92	2.74	1.46	0.81	1.92	2.74	.540
				*.09*	*.07*	*.14*	*.20*	*.09*	*.07*	*.14*	*.20*	
2	2.0			1.45	0.71	1.88	2.27	1.45	0.71	1.88	2.27	.794
				*.08*	*.07*	*.14*	*.16*	*.08*	*.07*	*.14*	*.16*	
3	24.1**	2.3	21.9	1.35	-0.73	0.58	2.84	1.08	-1.41	0.46	3.34	**.048**/.360
				*.10*	*.10*	*.09*	*.25*	*.10*	*.13*	*.12*	*.41*	
4	15.8*	0.1	15.7	1.52	-0.53	0.42	2.96	1.53	-0.48	0.70	2.84	**.000**/.202
				*.12*	*.09*	*.08*	*.26*	*.13*	*.08*	*.10*	*.30*	
5	11.9	0.1	11.8	1.92	-0.40	0.95	2.36	1.92	-0.40	0.95	2.36	.132
				*.10*	*.05*	*.06*	*.14*	*.10*	*.05*	*.06*	*.14*	
6	1.2			2.08	-0.62	1.04	2.49	2.08	-0.62	1.04	2.49	.746
				*.11*	*.05*	*.06*	*.14*	*.11*	*.05*	*.06*	*.14*	
7	6.3	0.7	5.6	2.47	0.01	0.98	2.38	2.47	0.01	0.98	2.38	.774
				*.13*	*.04*	*.05*	*.13*	*.13*	*.04*	*.05*	*.13*	
8	5.9	1.1	4.8	2.68	-0.03	1.50	2.58	2.68	-0.03	1.50	2.58	.686
				*.15*	*.04*	*.07*	*.15*	*.15*	*.04*	*.07*	*.15*	
9	95.0**	-0.0	95.0	2.03	0.41	2.26	3.12	2.04	1.14	2.70	3.79	.464 **/.052**
				*.17*	*.06*	*.16*	*.30*	*.26*	*.11*	*.31*	*.75*	
10	9.0	0.3	8.7	1.88	1.90	2.56	3.67	1.88	1.90	2.56	3.67	.718
				*.21*	*.13*	*.19*	*.37*	*.21*	*.13*	*.19*	*.37*	

*Note*.

1 Reported *p*-values are based on 500 bootstrap replications.

Table 5 reports the estimated parameters of the GRM which, for the DIF items, were obtained separately for males and females. Parameter estimates were obtained as follows. Firstly, the GRM was fitted to the eight locally independent items (*i.e*., items 3 to 8). Secondly, items 1 and 2 were scaled separately on the underlying latent attribute scale defined by the other eight items. This two-step procedure is justified by the result that *all *items had high loadings on the general factor of interest, as revealed in BFA and MSA (*i.e*., all items had *H_j_*≥ 0.3). The resulting item parameter estimates are unbiased because the eight items are fitted independently of the two locally dependent items, and items 1 and 2 are independently scaled on the underlying general attribute scale in the second step.

**Figure 2:**
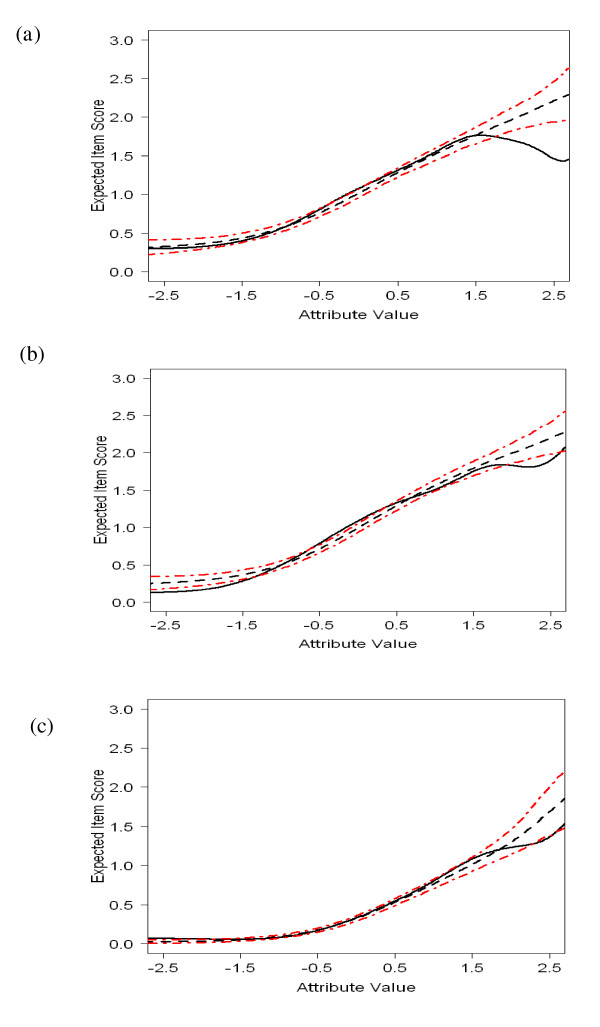
**Item-fit plots for (*a*) Item 3 (female sample), (*b*) Item 4 (female sample) and (*c*) Item 9 (male Sample)**. Figure note: Solid line = Observed item-score function; Dashed line = Expected item-score function under the Graded Response Model; Dashed/dotted lines indicate 95%variability envelopes under the Graded Response Model.

By constraining the parameters of the DIF-free items to be equal, we have item parameters that are on a common *θ*-scale. This property enables direct comparison of the psychometric properties of the EDS between males and females from the parameter estimates. To test the goodness-of-fit of the estimated GRM, we used a graphical approach proposed by Drasgow *et al. *[70] and a parametric bootstrap to test observed misfit for significance [*e.g*., [71]]. Items 3, 4, and 9 showed significant misfit (Table 5 column 13), whereas the other items fitted well. Figure 2 shows the item-fit plots for the three misfitting items. The solid lines are the observed item-mean score functions (IMSF) and the dashed lines are the expected item-mean score functions under the GRM. The red dashed-dotted lines display 95% variability envelopes, representing sampling fluctuations. If the solid line falls outside the 95% variability envelope, we have significant local misfit (two-tailed test, α = 0.05). Inspection of the plots showed that all three items misfitted at the extremes of the *θ *scale. Item 4 also showed misfit at *θ *ranges between -1 *< θ *< 1. However, the item-fit plots also showed that, at these ranges of the *θ *scale, the absolute deviance of the observed IMSF from the expected IMSF was small and is of no practical importance. In conclusion, a satisfactory fit was found with the GRM.

Inspection of the (unconstrained) *b *parameters for the DIF items (Table 5; columns 6 - 8 and 10 - 12) showed substantial differences in the thresholds for items 9 and 3 (ranging from 0.12 to 0.73). For example, the lowest threshold for item 9 was 0.41 for females and 1.14 for males. For item 4, the differences between estimated thresholds in males and females were small (ranging from 0.05 to 0.28).

**Figure 3:**
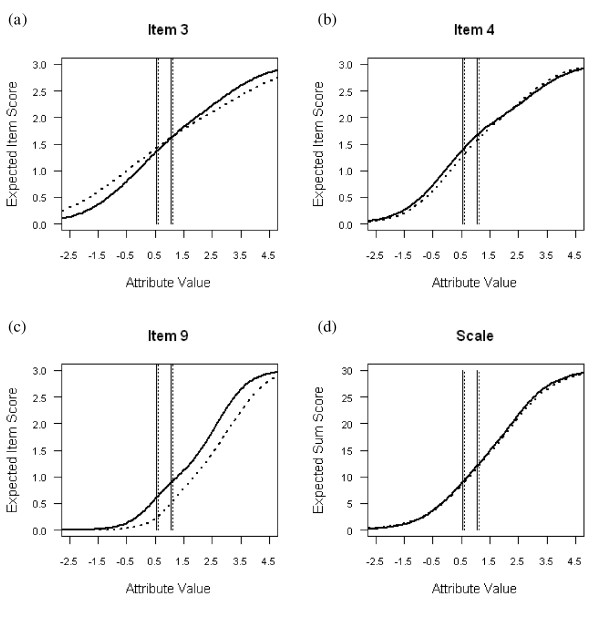
**Expected item-score functions for (*a*) Item 3, (*b*) Item 4, (*c*) Item 9, and (*d*) expected sum score as a function of the latent attribute (*θ*), for females (solid lines) and males (dashed lines)**.

To further study the impact of item bias, we plotted the expected item scores as a function of *θ *(see Figure 3A through 3C) for each of the three DIF items. In Figure 3 we also superimposed the cutoffs (vertical lines; solid lines for females, dashed lines for male s) that distinguish the diagnostic depression levels (to be explained below). For item 3 (Figure 3A) we found that at the higher end of the attribute scale males (dashed line) tended to report slightly lower levels of blaming oneself than females with the same attribute score (solid line), whereas the reverse was true at *θ *ranges below the cutoff point. Although DIF was significant, differences between expected scores for males and females due to DIF were too small (less than 0.27) to be of practical importance. For item 4 (Figure 3B), small differences of a maximum of 0.11 were found between the expected score for males and females at *θ *ranges of -1.5 to 2.0. For item 9 (Figure 3C), males were less likely to report that they had been crying than females, given equal depression levels. Maximum difference in the expected item scores due to DIF between males and females was 0.46. Finally, the expected sum score functions (Figure 3D) showed only minor differences between males and females. Positive and negative bias thus canceled each other out at the scale level. To summarize, noticeable gender bias was found for item 9 inquiring about crying behavior, but the DIF had little impact on gender-related bias in the sum scores.

### Results for Issue 3: What are the measurement properties of the EDS for screening depression?

**Figure 4:**
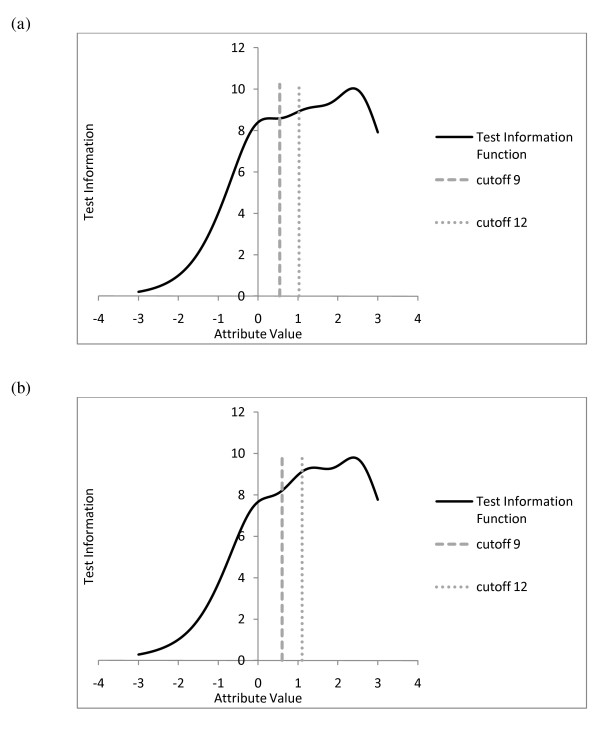
**Test information functions (solid lines) for (*a*) females and (*b*) males**. Figure note: Vertical lines represent clinical cutoffs for mild depression (dashed lines) and severe depression (dotted lines).

Since DIF was found for three EDS items, the psychometric properties will be examined separately for males and females when necessary, even though the impact of the DIF was quite small. Inspection of the estimated item parameters showed varying item discriminations across the items (Table 5, column 5 for females and column 9 for males). In particular, item 8 (*felt sad/miserable*) is the most discriminating item (*a *= 2.68) followed by item 7 (*difficulty sleeping*; *a *= 2.47), whereas item 3 (*blamed*) is the least discriminating item (*a*_female _= 1.35, *a*_male _= 1.08). Furthermore, the thresholds are located at the upper range of the latent attribute scale *θ*, implying that the items mainly differentiate respondents at higher ranges of the *θ*-scale. Figure 4 shows the total information functions for females and males. Once again, we see that the EDS is most informative at the higher ranges of the *θ *scale.

To evaluate the screening properties of the EDS and its constituent items in more detail, the cutoff scores on the *X*+ scale had to be translated into corresponding cutoffs on the *θ *scale (i.e., latent cutoffs). Since gender-related DIF appeared to be present in the data, different latent cutoffs were determined for females and males. For the cutoff score *X*_+ _= 9, the latent cutoff points were 0.54 for females and 0.60 for males. This means that a sum score of 9 on the EDS represents a somewhat higher depression level for males than for females. A result that is due to the DIF for item 9 on crying behavior. For the cutoff score *X*_+ _= 12, the corresponding latent cutoff points were 1.03 and 1.10 for females and males, respectively. This means that females with a *θ-*value in the range (0.54; 1.03), and males with a *θ-*value in the range (0.60; 1.10) exhibit symptoms of minor depression and require further clinical assessment, whereas males with *θ *> 1.10 and females with *θ *> 1.03 exhibit symptoms of major depression. The latent cutoff points are indicated by the vertical lines in Figures 3 and 4.

Inspection of the individual item contributions to the total information at the cutoff point (Table 6) showed that for distinguishing non-depression from mild depression, and mild depression from major depression, item 7 (*having difficulty sleeping*) is the most reliable indicator, followed by item 8 (*feeling sad/miserable*). The least reliable indicator for differentiating the diagnostic groups is item 10 (*thought of self harm*).

**Table 6:** Individual item contribution to the test information function at cutoffs, for females and males

		Females		Males	
		
		*X*_+ _= 9	*X*_+ _= 12	*X*_+ _= 9	*X*_+ _= 12
Item	Item Label	(*θ *= 0.54)	(*θ *= 1.03)	(*θ *= 0.60)	(*θ *= 1.10)
1	laugh	6%	7%	7%	7%
2	enjoyment	6%	7%	7%	7%
3	blamed	6%	5%	4%	3%
4	anxious/worried	7%	6%	8%	7%
5	scared/panicky	12%	11%	12%	11%
6	things get on top of me	12%	13%	13%	13%
7	difficulty sleeping	19%	19%	20%	18%
8	sad/miserable	16%	16%	16%	17%
9	crying	12%	10%	10%	12%
10	thought of self harm	3%	5%	3%	6%

**Figure 5:**
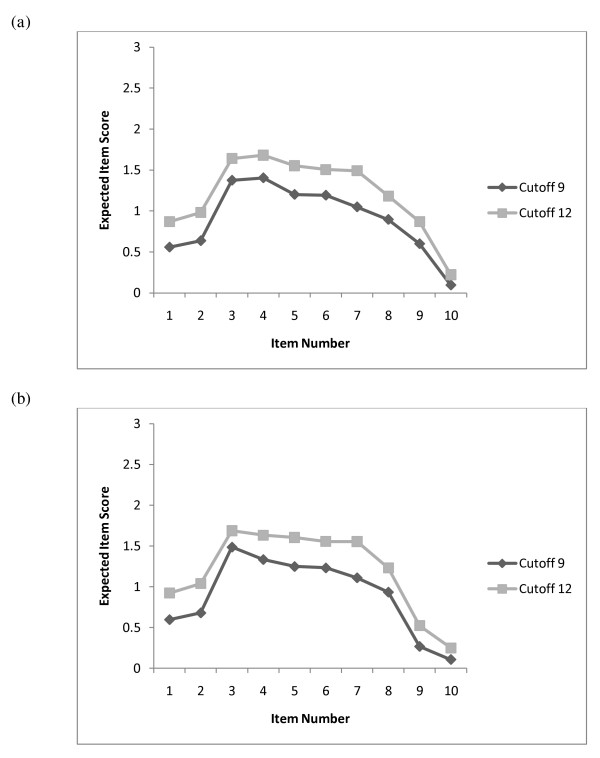
**Expected item-score profiles, for (*a*) females and (*b*) males, at the latent cutoff points differentiating diagnostic groups of non-depressed, mildly depressed, and severely depressed**.

Figure 5 shows the item-score profiles for females and males at a particular point on the *θ *scale and can be conceived as the (expected) item means in a sample of respondents with a *θ *value equal to the cutoff. Comparing the profiles at the cutoffs shows the greatest differences between item 7 (sleeping difficulties) and items 1 and 2 (hedonia), followed by items 5 and 6. This suggests that the difference between mild and severe depression typically expresses itself to a greater extent by a decrease in hedonistic thoughts and an increase in sleeping problems, and to a lesser extent by an increase in feelings of panic and the feeling that things have been getting on top of oneself.

## Discussion

In the present study we investigated the dimensionality and scaling properties of the EDS in a population of patients with DM2. The objectives of this study were threefold. Firstly, we examined the dimensionality of the EDS. An important practical and scientific issue is whether the items, each covering different conceptually narrow aspects (*e.g*., anxiety, dysphoria, and anhedonia), together constitute a proper unidimensional scale for measuring the general broad attribute of depression. Measurements of the general attribute, covering the full breadth of the construct, may have higher predictive validity than subscale scores [33]. Confirmatory factor analyses and Mokken scale analysis showed that the ten EDS items constitute a unidimensional scale for the general depression factor of interest. Respondents can be reliably ordered on this dimension using the sum score. These results justify the use of sum scores on the EDS as measurements of the underlying depression attribute. This finding corroborates the original intentions of the developers, who designed the EDS to be unidimensional [26].

The question of whether a set of items covering different aspects should be treated as one unidimensional scale for the general attribute of interest, or should be divided into smaller subscales, is an important issue in psychological assessment. Our study demonstrated that both the nonparametric IRT framework (as an exploratory approach) and bifactor models (as a confirmatory approach) provide powerful tools for rigorously examining to what extent the items in a scale together measure a broad attribute in the presence of specific aspects. Typically, a general attribute dimension is present if MSA clusters all items into a single scale for medium values of lower bound *c*, and yields separate clusters for high values of *c*, and if all items have high loadings on the general factor in the bifactor model. In some instances, however, the existence of a general underlying attribute is easily derived from the factor solutions themselves. For example, De Bruin *et al. *[30] tested the two-factor model of Pop *et al. *[32] in a confirmatory factors analysis, and found a correlation of .86 between the two factors. From this high correlation, they concluded that the factors basically provide information about the same underlying construct. Such compelling evidence is the exception rather than the rule.

The dimensionality analyses revealed small local dependencies between items 1 and 2. We hypothesize that local dependence can be explained by the opposite wording compared to the other items in the EDS. Local dependencies related to item wording are typical for scales comprising negatively- and positively-worded items [72]. The literature provides competing explanations as to why these additional dependencies often emerge in balanced scales. One explanation states that positively-worded items are dimensionally distinct from negatively- worded ones. For example, being *unhappy *is different from *not being happy*. Other explanations include careless responding [73] and carry-over effects due to similarity in wording [72]. Whether the two items should be regarded as covering a dimensionally distinct attribute or as being caused by idiosyncratic response tendencies or wording effects is difficult to tell from a single data analysis. Future research may explore a modified version of the EDS in which all the items are worded in the same direction, to see whether local dependence vanishes.

Another scale refinement that may be pursued in future research is to remove the locally-dependent items 1 and 2 and use an eight-item version of the EDS. However, given the results of our study, we believe that removing items 1 and 2 is not to be recommended. MSA, CFA, and parametric IRT analyses consistently showed that the two items are reliable indicators of the general attribute. In addition, bifactor analyses showed that the bias in estimated scale reliability was only 0.01. Thus, from a pure practical point of view, ignoring local dependence does not impair the valid use of EDS scores. Removing the items, however, would result in a loss of information and would compromise the reliability and increase the risks of incorrect diagnosis. The two-step estimation approach adopted in our study facilitates forecasting the consequences of removing items 1 and 2 from the EDS. For example, the two items accounted for 12% to 14% of the information around the cutoff. Removing these items reduces the test information around the cutoff by a factor 1.2. In addition, removing items 1 and 2 reduces Cronbach's alpha from 0.86 to 0.82 (results based on a simulated data set of 10,000 item-response vectors; details available from the second author). This may seem small, but it should be noted that decreasing reliability caused by test length has several adverse effects, including a reduction in the power to find group differences, additional bias in the estimated regression effects of the EDS, and higher risks of classification errors (*e.g*., [74]). Furthermore, removal of the items necessitates determining new cutoffs for diagnosing mild and severe levels of depression, and may unduly narrow the construct since one aspect (anhedonia) may no longer be well represented.

The second objective of this study was to test whether the EDS is biased with respect to gender. Significant DIF was found for items 3 (*blaming*), 4 (*anxious*) and 9 (*crying*), but only for item 9 did DIF lead to appreciable differences in expected responses for males and females. However, at the scale level, the presence of DIF caused no substantial differences in the expected scores between males and females. The minor impact of DIF was also evident from the small differences between latent cutoffs that were obtained separately for males and females. For example, for the screening for mild depression we found latent cutoffs of *θ *= 0.54 and *θ *= 0.60 for females and males, respectively. Such a difference is negligible given that *θ *is standard normally distributed. Altogether these findings indicate that the observed DIF had no practical impact, and justify using the same screening rules for males and females.

The third objective was to have a more detailed picture of the screening properties of the EDS items. We found that the EDS is only informative at the higher ranges of the *θ *scale. This is a common result for many clinical scales [43], which basically assess symptom severity with respect to a clinical condition (*e.g*., depression). This means that the items in the scale only assess one polar of the 'no depression-depression' continuum and constitute a quasi- attribute [43]. Secondly, we found that, for the distinction between no depression and mild depression and for that between mild depression and severe depression, item 7 (*difficulty sleeping*) appeared to be the most reliable indicator, followed by item 8 (*sad/miserable*). For the other items, differences in the relative contribution to the information between the two cutoffs were also small, which means that for differentiating respondents around the higher cutoff (*X*_+ _= 12), the relative importance of the items is the same as for differentiating around the lower cutoff (*X*_+ _= 9). In addition, the differences in screening properties between males and females were small, which again demonstrates that the impact of DIF is small and of no practical concern. Thirdly, we looked at the score profiles that further characterize the diagnostic groups at the item level. We found that the difference between mild and severe depression is most prominently reflected by differences in sleeping difficulties and anhedonia.

In this study, we used IRT-based methods to examine different aspects of the EDS. To the best of our knowledge, there are two other studies that have used IRT to validate the EDS [48,49]. Both those studies adopted a polytomous Rasch model (*e.g*., [42,55]), which assumes, for example, that all the items in a scale have the same discrimination power. This assumption is unrealistic for the EDS, as shown by the varying item-factor loadings in the factor analysis and the varying scalability coefficients in Mokken scale analysis. Therefore, the Rasch model seems to be too restrictive to adequately capture the relevant test and item characteristics of the EDS. Using an IRT model that is too restrictive yields undesirable results. Most importantly, it may lead to the removal of sound items. For example, Pallant *et al. *([48], *p*. 28) suggested discarding item 8 from the EDS because it showed poor fit under the postulated Rasch model. However, this misfit is most likely explained by the fact that the item has higher discrimination than the other items. Under the Rasch model, such deviating item discrimination is identified as item misfit. Discarding item 8 seems to be an unfortunate choice since, as was shown in this study, it is highly informative for diagnosing mild and severe depression levels and has excellent measurement properties. Removing item 8 would unnecessarily compromise the reliability and (predictive) validity of the EDS.

Although the above findings support the dimensionality and reliability of the EDS, two limitations should be noted. Firstly, we limited our study to analysis of the dimensionality and measurement properties of the EDS. However, for an instrument to be a valid screening tool in patients with an elevated risk of adverse health outcomes, additional studies on the sensitivity and specificity must also be carried out. The sensitivity and specificity of the EDS have been extensively studied in pregnant [75], non-postnatal [27], and menopausal-aged [76,77] women. Unfortunately, we had no information on clinical diagnoses derived from psychiatric diagnostic interviews at our disposal. Data from a psychiatric diagnostic interview such as the Composite International Diagnostic Interview would allow us to calculate the sensitivity and specificity of the Dutch version of the EDS for primary-care patients with type 2 diabetes.

The second limitation concerns the specific sample of diabetes patients used in our study. Published validation studies on other scales measuring depressive symptoms, such as the HADS [78] and the SCL-90-R [79], have sometimes yielded inconsistent results with respect to the dimensionality of the scales across different (clinical) populations. These inconsistencies can partly be explained on statistical grounds since researchers use different research strategies and model selection criteria [80]. However, it has also been hypothesized that the dimensionality of symptom scales may depend on the general level of negative affectivity - a concept closely related to depression - itself [80]. This means that for a well- defined population, the dimensionality within the subpopulation with high negative affectivity may be different from within the subpopulation with low negative affectivity. According to this hypothesis, negative affectivity serves as a so-called *structure generating factor*. However, not only the general negative affectivity level but also specific characteristics of the disease status of the respondents may operate as a structure-generating factor. This means that caution must be exercised in generalizing the results from one clinical population to another.

## Conclusions

Dimensionality and scale analysis in a large sample of 1,656 males and females diagnosed with type 2 diabetes suggest that the ten EDS items constitute a psychometrically sound scale, representing a broad depression attribute. The EDS can be safely used as a valid and reliable screening tool for both males and females with type 2 diabetes, using the same cutoff values to define categories of patients with mild and severe levels of depressive symptoms.

## Abbreviations

AISP: automatic item selection procedure; BFA: Bifactor Analysis; CFA: confirmatory factor analysis; DIF: differential item functioning; DM2: type 2 diabetes mellitus; EDS: Edinburgh Depression Scale; EFA: exploratory factor analysis; GRM: graded response model; IRF: item response function; IMSF: item-mean score function; MHM: monotone homogeneity model; MSA: Mokken scale analysis.

## Competing interests

The authors declare that they have no competing interests.

## Authors' contributions

**EdC **participated in data preparation, statistical analysis, writing the manuscript, and designing the tables; **WE **participated in statistical analysis and writing the manuscript; **GN **participated in data collection and preparation, and helped in writing the manuscript; **VP **participated in the design of the study, and helped in writing the manuscript; **FP **participated in the design of the study, and helped in writing the manuscript. All the authors have read and approved the final manuscript.

## Pre-publication history

The pre-publication history for this paper can be accessed here:

http://www.biomedcentral.com/1471-244X/11/141/prepub
